# Open-Source Data Analysis Tool for Spectral Small-Angle X-ray Scattering Using Spectroscopic Photon-Counting Detector

**DOI:** 10.3390/s24165307

**Published:** 2024-08-16

**Authors:** Sabri Amer, Andrew Xu, Aldo Badano, Eshan Dahal

**Affiliations:** Division of Imaging, Diagnostics, and Software Reliability, Office of Science and Engineering Laboratories, Center for Devices and Radiological Health, Food and Drug Administration, Silver Spring, MD 20993, USA

**Keywords:** photon-counting detector, data analysis tool, spectral SAXS, X-ray scattering, material characterization

## Abstract

Spectral small-angle X-ray scattering (sSAXS) is a powerful technique for material characterization from thicker samples by capturing elastic X-ray scattering data in angle- and energy-dispersive modes at small angles. This approach is enabled by the use of a 2D spectroscopic photon-counting detector that provides energy and position information of scattered photons when a sample is irradiated by a polychromatic X-ray beam. Here, we describe an open-source tool with a graphical interface for analyzing sSAXS data obtained from a 2D spectroscopic photon-counting detector with a large number of energy bins. The tool takes system geometry parameters and raw detector data to output 1D scattering patterns and a 2D spatially-resolved scattering map in the energy range of interest. We validated these features using data from samples of caffeine powder with well-known scattering peaks. This open-source tool will facilitate sSAXS data analysis for various material characterization applications.

## 1. Introduction

When a polychromatic X-ray beam passes through a material, some X-ray photons are absorbed and scattered in the interaction process in an energy-dependent manner. The spectral SAXS (sSAXS) technique captures elastically scattered X-ray photons simultaneously in angle- and energy-dispersive modes at small angles (less than $10^{\circ }$) to identify materials [1]. The scattering signature of the material is analyzed in reciprocal space (q-space) for material identification. Traditional SAXS collects q-data in angle-dispersive mode, where the scattering signal is measured as a function of the scattering angle at fixed energy using monochromatic X-rays. Energy-dispersive SAXS (EDSAXS) commonly measures the scattering signal as a function of energy for a fixed angle. EDSAXS employs a polychromatic X-ray source to irradiate the sample. An energy-sensitive detector is placed at a constant scattering angle to record scattered X-ray energy. While angle-dispersive SAXS has a high angular resolution, EDSAXS offers a faster data acquisition time to access the same q-range. Schultz and Long [2] and Bordas et al. [3] conducted pioneering work on EDSAXS in the 1970s. Bordas et al. obtained the energy-dispersive scattering pattern from rat-tail collagen in 200 s [3]. The angular acceptance of the detector was defined by placing a lead collimator in front of the detector. Various experimental approaches to EDSAXS and its application have been reported since then [4,5,6,7]. The major drawback of EDSAXS is low q-resolution. With low q-resolution, we may not be able to resolve sharp peaks at well-defined q-values.

Over the last decade, semiconductor-based photon-counting detectors have increasingly advanced to record energy and position information of each X-ray photon in a wide energy range with a high number of energy bins and smaller pixel sizes [8,9,10]. For instance, a cadmium telluride (CdTe)-based pixellated detector called HEXITEC has shown promising results in X-ray diffraction studies for detecting explosives and drugs [11,12]. In these studies, a 2D spectroscopic detector was used to collect full energy spectrum and scattering angle information in each pixel. At a basic level, such a 2D spectroscopic detector consists of a single CdTe crystal with electrodes and an application-specific integrated circuit (ASIC) [13]. X-rays enter through a planar cathode to interact with the CdTe. An anode is uniformly divided into pixels and connected to ASIC via bump bonds for pixel readout. An ASIC processes the pixel charge collected in each photon interaction to recover the energy information. We can capture the angular distribution and energy information of scattered photons across pixels without moving the detector or using collimators. However, the performance of 2D photon-counting detectors is not perfect. There are various phenomena when detecting X-ray photons that cause the degradation of measured photon counts and energy spectra [14,15]. The pulse pileup and charge sharing are two major problems that will degrade the performance of a 2D spectroscopic detector, especially when using high X-ray flux and small pixel geometries.

Moss et al. have recently utilized the HEXITEC detector in angle- and energy-dispersive XRD studies to record scattering patterns from breast tissue samples and use them to discriminate between normal and cancerous tissue types [16]. The sSAXS method takes advantage of these spectral detectors to collect angle- and energy-resolved data at small angles below $10^{\circ }$ using high-energy polychromatic X-rays. This allows not only reducing the measurement time utilizing the available X-ray flux from a polychromatic X-ray source without spectrum filtration, but also probing thicker samples in the cm scale using a high energy range of interest to maximize elastic scattering. To date, sSAXS has been applied for various material characterization applications from identifying embedded materials in thick samples [1] to assessing the protein accumulation in neurodegenerative brains [17]. As photon-counting detectors with a high number of energy bins become more widely used, there is a need for a user-friendly data analysis tool to process the detector data with the goal to leverage spectral information in sSAXS studies.

In this paper, we describe an open-source tool with a graphical interface designed for analyzing sSAXS data obtained from a 2D spectroscopic photon-counting detector with a large number of energy bins to enable material characterization applications. The tool takes system geometry parameters and 3D raw detector data, which include energy and position information of every recorded count, to output 1D scattering patterns and a 2D spatially resolved scattering map in the energy range of interest.

## 2. Materials and Methods

### 2.1. Typical Setup for Data Collection

**Figure 1:**
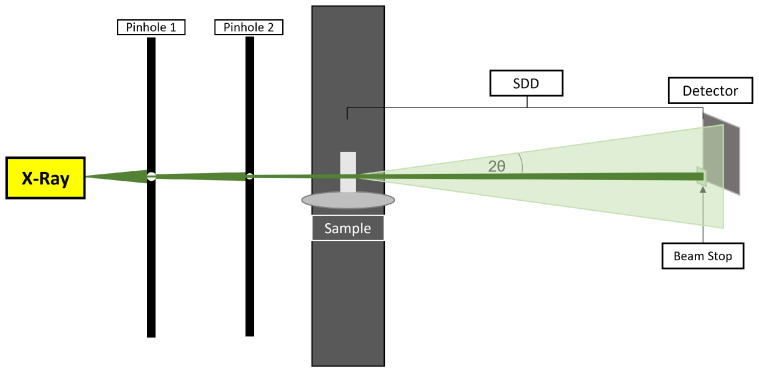
Schematic of a typical sSAXS setup used to collect elastic scattering data in angle- and energy-dispersive modes at small angles.

The schematic of a typical sSAXS setup for collecting data is shown in Figure 1. The sSAXS system includes a dual-pinhole collimated polychromatic X-ray beam irradiating the sample. A 2D spectroscopic detector is placed at a certain sample-to-detector distance (SDD) to collect scattered photons at small angles. The SDD is in the 220 to 300 mm range to cover the q-range of interest. The primary beam is blocked by the beam stop to avoid spectral degradation at high flux. Caffeine data were collected from a previously validated prototype sSAXS system, as reported in detail elsewhere [1,17]. Briefly, we used the MXR-160/22 industrial X-ray tube (tungsten anode, COMET) to generate a polychromatic X-ray beam. The polychromatic X-rays were generated using a tube voltage of 50 to 80 kVp and a current of 1 mA. The generated beam was collimated using two lead pinhole collimators with diameters of around 2 mm to create a pencil beam while decreasing parasitic scattering. The HEXITEC detector (Quantum Detectors Ltd, Oxfordshire, UK) was used to record energy and position information of scattered photons in an 80 × 80 pixels sensor with 200 energy bins and 1 keV energy binning. The charge sharing discrimination algorithm was used for a charge sharing correction to remove shared events from the nearest neighboring pixels [18].

### 2.2. Detector Data Analysis

**Figure 2:**
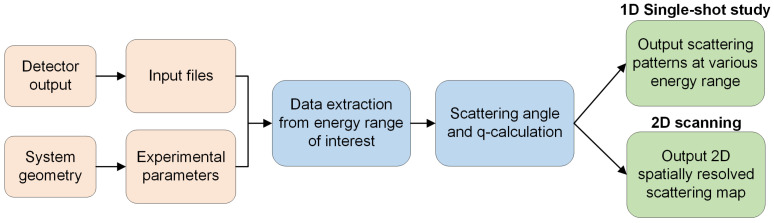
Overall data analysis workflow implemented in the software.

Figure 2 shows the overall data analysis workflow implemented in the software. The analysis begins by taking the system geometry information and raw detector data in the energy range of interest as an input. The raw detector data have count and energy information of the scattered photons in each pixel that can be stored in a three-dimensional array, where the first index specifies the energy bin and the second and third indices represent the X, Y location of the pixel. With these raw values extracted, the data outside of the energy range of interest can be removed. The scattered photons in each pixel have a known scattering angle (2θ) based on the system geometry (Figure 1) with a known SDD and transmitted primary beam location. With known energy *E* and scattering angle 2θ, the momentum transfer *q* is calculated as follows:

(1) $$q=\frac{4\pi E\sin\theta }{hc},$$

where hc=1.24 keV nm. The number of occurrences of each *q* value are then binned to produce the final summed q-data as an output. For a 2D scanning experiment, the user can point the program to the folder containing the individual measurements to output a 2D spatially resolved scattering map based on the area under the peak (AUP) values in the q-range of interest. Since there is no spatial information inherently present in the detector files, the 2D map is dependent on the user specifying the dimensions of the image they want based on how the samples were scanned. If a background measurement has been taken for subtraction, the user may specify the file and have its signal removed from all other collected measurements.

### 2.3. User Interface and Inputs

**Figure 3:**
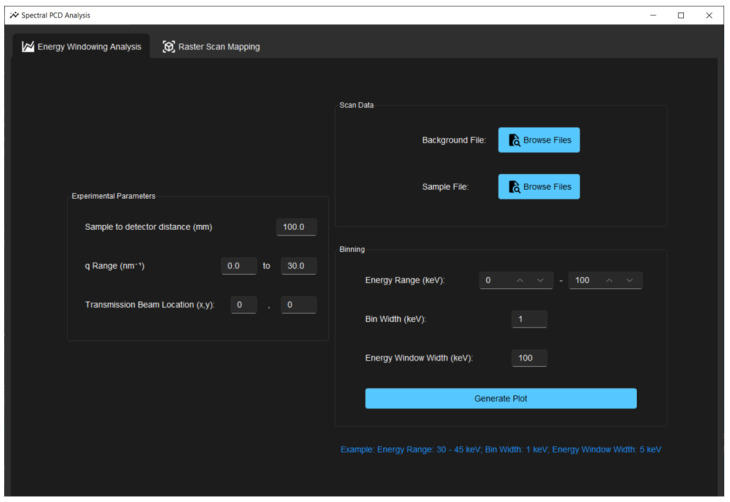
User interface of the program for inputs to generate scattering plots and a 2D scattering map in the energy range of interest.

The script runs as an independent executable built in Python. The user interface, as shown in Figure 3, was built using the Tkinter library. On start-up, the script prompts the user for the data files, experimental parameters from the system geometry, and the energy range of interest. The total energy range can be arranged into energy bins and energy windows, where energy windows are subdivisions of the entire energy range. For example, if the user wishes to look at one window across the range of 30–50 keV with data points every 4 keV, they would specify this to the program by inputting the following arguments: binStart = 30 keV, binEnd = 50 keV, binWidth = 4 keV, energyWindow = 20 keV. Test data from a sample of pure caffeine powder were used to evaluate the program, as caffeine is known to have Bragg peaks around 8.4 and 18.6 nm^−1^. Note that the data used for validation are limited to data collected from the HEXITEC detector, but the tool can be be used or modified with any detector with similar file format, where the counts and energy information in each pixel is stored in a three-dimensional array.

## 3. Results and Discussion

**Figure 4:**
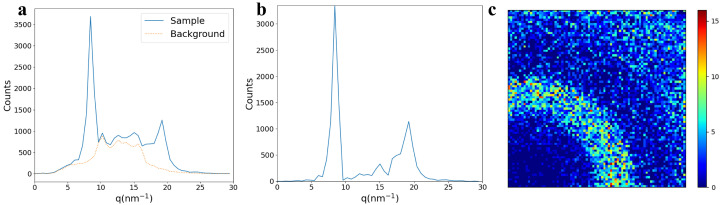
Generated plots using caffeine data for the validation of the software. (**a**) Count as a function of *q* using sample data from caffeine powder and background data from the sample holder. (**b**) Background-subtracted 1D scattering pattern of caffeine sample, showing its known Bragg peaks at the correct q-position around 8.4 and 18.6 nm^−1^. (**c**) Corresponding 2D detector data showing background-corrected counts in each pixel.

Outputs from the tool are described here with the goal of validating and showcasing its data analysis capabilities using the caffeine data. Figure 4 shows the generated plots with the scattering patterns of caffeine having the two Bragg peaks around 8.4 and 18.6 nm^−1^, as reported before [1]. The photon count as a function of *q* is plotted to show the 1D scattering data before and after background subtraction in Figure 4a,b, respectively. The background-subtracted 2D detector data for caffeine is shown in Figure 4c to visualize the recorded scattering pattern with counts per pixel information. The peaks at 8.4 nm^−1^ and 18.6 nm^−1^ correspond to the atomic spacing of caffeine reported around 7.53–7.40 Å and 3.37–3.30 Å, respectively [8]. While this confirms that the sSAXS setup accurately captures scattering information, it is necessary to perform transmission and thickness corrections if the samples have non-uniform thickness and relatively high attenuation. It is also important to study linearity in recorded counts with thickness to understand the detector response. The high count rate may degrade the detector performance due to pulse pileup. When several overlapping pulses are generated by quasi-coincident photons, especially with high X-ray flux, they may pile up and be detected as one pulse. Due to this pulse pileup phenomenon, the detector may record a singe count for multiple photons with incorrect energy.

**Figure 5:**
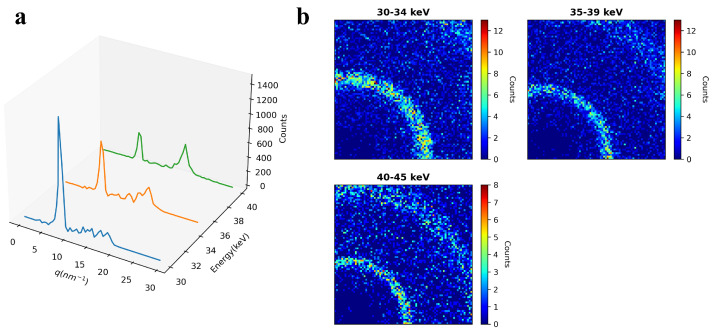
Demonstration of energy windowing capability. (**a**) The q-data of the caffeine separated into three energy windows. (**b**) The corresponding 2D detector data showing counts per pixel separated into three energy windows defined by the user.

Figure 5 demonstrates the energy windowing capability of the tool. The 1D scattering pattern and 2D detector data are plotted across three energy windows defined by the user in the 30 to 45 keV energy range. Higher q-data are covered with an increase in the energy based on the *q* and *E* relation in Equation (1). Therefore, the second ring in the 2D detector data (Figure 5b) becomes more visible with an increase in the energy. The user can change the energy window size in the input as needed to analyze data in various energy windows within the full energy range covered by the detector [19]. We used 1 keV energy binning to collect the raw data to have more flexibility in choosing narrower or wider energy windows depending on the materials or scattering features of interest. The q-data per energy windows can always be summed together with q-binning to combine the scattering information present at each *E*. We selected the energy range above 30 keV to probe thicker samples and capture scattering peaks of caffeine while avoiding signal contamination from the fluorescence peaks of Cd and Te in the CdTe-based photon-counting detector. It is up to the user to experimentally analyze and select the energy range of interest depending on the material to maximize elastic scattering information. We usually employ Monte Carlo simulations to understand the scattering contributions from coherent (elastic), Compton, and multiple scattering with energy and thickness for the sample under study. This approach allows for systematically studying the interaction effects and selecting the energy range that maximizes elastic scattering while minimizing other degrading factors related to multiple scattering and detector performance.

**Figure 6:**
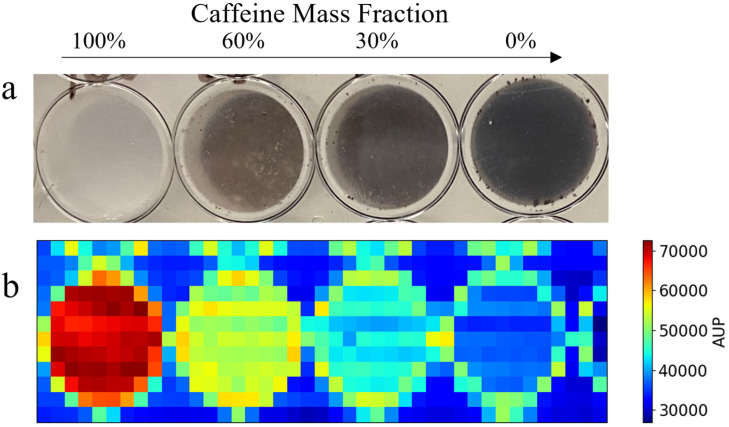
Two-dimensional spatially resolved scattering map output using raster scan data. (**a**) A well plate sample containing four distinct mass fractions of caffeine. (**b**) The corresponding scattering map, generated using the area under the peak (AUP) values from 7 to 9 nm^−1^. The AUP q-range is defined by the user in the input to use the first peak of caffeine to create the planar image.

Furthermore, our tool has the capability of outputting a 2D scattering map using scanning data as input. Figure 6a shows a picture of a well plate containing four different mass fractions of caffeine powder that was raster-scanned to collect the scanning sSAXS data. The uploaded scanning data in the program generated the spatially resolved scattering map based on the AUP values from 7 to 9 nm^−1^, as shown in Figure 6b. The user defines the q-range to calculate the area under the peak values to generate this planar image. In this case, we chose 7 to 9 nm^−1^ to calculate AUP values from the first peak of caffeine. We observe a high scattering effect around the circular edge of the well. There is a thin circular ring with high AUP values around each well in the plastic plate. Nevertheless, the scattering map, based on the AUP values, visually correlates with the mass fraction of the caffeine powder in each well. There is no direct energy windowing capability in the 2D scatter map. However, the user can pre-select the energy range while inputting the spectroscopic detector data for analysis. This allows the user to plot the same data over multiple different energy ranges, providing flexibility in energy discrimination and enabling a more detailed analysis of the scatter map data. It is advantageous to use this scanning approach to differentiate materials based on their scattering properties. However, the disadvantage is the long scanning time required to cover even a small area due to the use of a pencil beam for point-by-point scanning. Therefore, a pencil beam scan is naturally suited for region-of-interest analysis, which requires high spatial and q-resolution. The trade-off between resolution and scanning time should be considered when employing this scanning approach.

In our previous work, we extensively used the prototype sSAXS system to collect sSAXS data from diverse sample types, ranging from weakly scattering proteins [17] to highly scattering polymethyl methacrylate (PMMA) material [1]. The current version of the tool is designed to handle a wide range of samples effectively, provided that users supply the necessary file for background subtraction without significant variation in thickness and attenuation properties. We have deposited our previously collected data from plasma protein and PMMA on GitHub to help end users understand the tool’s capability to handle various sample types. Our tool is better suited for the material characterization of solid samples with materials that have some degree of crystallinity or a repeating molecular structure. This is because of the ease of detecting strong scattering features or Bragg peaks in the accessible q-range defined by system geometry and the energy used. We used polycrystalline caffeine samples with known d-spacing information to validate the tool, where the peak position is related to the d-space by 2π/d.

## 4. Conclusions

We described a tool for analyzing single-shot and scan sSAXS data obtained from a 2D spectroscopic photon-counting detector. Its features were validated using data from samples of caffeine powder with well-known scattering peaks. The tool is open-source and freely available at https://github.com/DIDSR/Spectral-SAXS-Analysis-Tools (accessed on 5 August 2024). Future improvements to this work include adding various data correction steps to accurately compare scattering data across different measurement settings, system geometries, and sample thicknesses. This tool can be used as is with a simple background correction or modified to incorporate other detector data formats and data correction methods for various X-ray scattering- or diffraction-based material characterization applications.

## Data Availability

Data available at https://github.com/DIDSR/Spectral-SAXS-Analysis-Tools (accessed on 5 August 2024).
